# Evaluation of High-Deductible Health Plans and Acute Glycemic Complications Among Adults With Diabetes

**DOI:** 10.1001/jamanetworkopen.2022.50602

**Published:** 2023-01-20

**Authors:** David H. Jiang, Jeph Herrin, Holly K. Van Houten, Rozalina G. McCoy

**Affiliations:** 1 Division of Health Care Delivery Research, Robert D. and Patricia E. Kern Center for the Science of Health Care Delivery, Mayo Clinic, Rochester, Minnesota; 2 Section of Cardiovascular Medicine, Department of Internal Medicine, Yale School of Medicine, New Haven, Connecticut; 3 Division of Community Internal Medicine, Geriatrics, and Palliative Care, Department of Medicine, Mayo Clinic, Rochester, Minnesota; 4 OptumLabs, Eden Prairie, Minnesota

## Abstract

**Importance:**

Optimal diabetes care requires regular monitoring and care to maintain glycemic control. How high-deductible health plans (HDHPs), which reduce overall spending but may impede care by increasing out-of-pocket expenses, are associated with risks of severe hypoglycemia and hyperglycemia is unknown.

**Objective:**

To examine the association between an employer-forced switch to HDHP and severe hypoglycemia and hyperglycemia.

**Design, Setting, and Participants:**

This retrospective cohort study used deidentified administrative claims data for privately insured adults with diabetes from a single insurance carrier with multiple plans across the US between January 1, 2010, and December 31, 2018. Analyses were conducted between May 15, 2020, and November 3, 2022.

**Exposures:**

Patients with 1 baseline year of enrollment in a non-HDHP whose employers subsequently forced a switch to an HDHP were compared with patients who did not switch.

**Main Outcomes and Measures:**

Mixed-effects logistic regression models were used to examine the association between switching to an HDHP and the odds of severe hypoglycemia and hyperglycemia (ascertained using diagnosis codes in emergency department [ED] visits and hospitalizations), adjusting for patient age, sex, race and ethnicity, region, income, comorbidities, glucose-lowering medications, baseline ED and hospital visits for hypoglycemia and hyperglycemia, and baseline deductible amount, and applying inverse propensity score weighting to account for potential treatment selection bias.

**Results:**

The study population was composed of 42 326 patients who switched to an HDHP (mean [SD] age: 52 [10] years, 19 752 [46.7%] women, 7375 [17.4%] Black, 5740 [13.6%] Hispanic, 26 572 [62.8%] non-Hispanic White) and 202 729 patients who did not switch (mean [SD] age, 53 [10] years, 89 828 [44.3%] women, 29 551 [14.6%] Black, 26 689 [13.2%] Hispanic, 130 843 [64.5%] non-Hispanic White). When comparing all study years, switching to an HDHP was not associated with increased odds of experiencing at least 1 hypoglycemia-related ED visit or hospitalization (OR, 1.01 [95% CI, 0.95-1.06]; *P* = .85), but each year of HDHP enrollment did increase these odds by 2% (OR, 1.02 [95% CI, 1.00-1.04]; *P* = .04). In contrast, switching to an HDHP did significantly increase the odds of experiencing at least 1 hyperglycemia-related ED visit or hospitalization (OR, 1.25 [95% CI, 1.11-1.42]; *P* < .001), with each year of HDHP enrollment increasing the odds by 5% (OR, 1.05 [95% CI, 1.01-1.09]; *P* = .02).

**Conclusions and Relevance:**

In this cohort study, employer-forced switching to an HDHP was associated with increased odds of potentially preventable acute diabetes complications, potentially because of delayed or deferred care. These findings suggest that employers should be more judicious in their health plan offerings, and health plans and policy makers should consider allowing preventive and high-value services to be exempt from deductible requirements.

## Key Points

**Question:**

Does an employer-mandated switch to a high-deductible health plan (HDHP) increase the risks of severe hypoglycemia and hyperglycemia among patients with diabetes?

**Findings:**

In this cohort study of 245 055 US adults with diabetes enrolled in employer-sponsored health plans, forced transition to an HDHP increased the odds of experiencing an emergency department (ED) or hospital visit for severe hyperglycemia by 25% during the study period, with the odds increasing 5% for each year of HDHP enrollment. Although forced transition to HDHP did not increase the odds of ED and/or hospital visits for severe hypoglycemia over the whole observation period, the odds increased by 2% for each year enrolled in an HDHP.

**Meaning:**

In this study, patients with diabetes who switched to HDHPs were significantly more likely to experience serious, but preventable, acute diabetes complications compared with patients who remained in conventional insurance plans; these findings suggest the need for policy solutions to address health plan-mediated barriers to diabetes care.

## Teaser

This cohort study examines the association between an employer-forced switch to a high-deductible health plan (HDHP) and severe hypoglycemia and hyperglycemia among adults with diabetes.

## Introduction

Diabetes is one of the most costly chronic health conditions for both patients and society, accounting for approximately 1 in every 4 U.S health care dollars.^1^ Because people with diabetes incur high out-of-pocket expenditures for their care,^1^ availability and type of health insurance can have substantial impacts on diabetes management and health outcomes. Underinsurance in the setting of high-deductible health plans (HDHPs), defined for 2020 through 2022 as plans with individual deductibles of at least $1400, has become increasingly widespread as employers sought less costly ways to provide the minimal essential coverage required by the Patient Protection and Affordable Care Act and as individual enrollments in Marketplace plans increased.^2,3,4^ By 2020, nearly 30% of individuals with employer-sponsored health plans had HDHPs.^5^ As HDHP popularity continues to rise, it is essential to understand its impact on diabetes management and outcomes.

Although many employers offer a range of health plans to their employees, including both HDHP and non-HDHP options, some offer only HDHP. Because HDHP enrollees must pay in full for health expenses until their annual deductible is met, they may be pressured to delay or forego care, particularly for asymptomatic but costly conditions such as diabetes. Prior research has found that HDHP enrollment among patients with diabetes is associated with increased financial burden,^6,7^ less frequent ambulatory care,^8^ slightly lower medication adherence,^9^ increase in low-severity emergency department (ED) visits,^10^ and delayed care for cardiovascular conditions,^11^ albeit with no apparent change in the rates of incident cardiovascular events.^12^ These detrimental effects were most apparent among patients with lower income levels.^7,8,10^ Evidence about HDHP’s effect on glycemic control and acute diabetes complications, however, is scarce.^13^

To test our hypothesis that HDHP enrollment is associated with increased risks of experiencing severe dysglycemia, we examined the rates of ED visits and hospitalizations for severe hypoglycemia and hyperglycemia (ie, diabetic ketoacidosis and hyperglycemic hyperosmolar state) among adults with diabetes who were forced to switch from non-HDHP to HDHP. We hypothesized that patients will be more likely to experience severe dysglycemic events after forced transition to HDHP and that the effect size would increase for each year of HDHP enrollment due to cumulative financial strain on the patient.

## Methods

### Data Source and Study Design

In this cohort study with stepped wedge design (see eMethods in Supplement 1 for details), we analyzed medical and pharmacy claims data of enrollees in employer-sponsored health plans included in OptumLabs Data Warehouse (OLDW), a large administrative claims database composed of health plans offered by a single large nationwide insurance company.^14^ All data were accessed after statistical deidentification; and the Mayo Clinic institutional review board deemed the study exempt from review and the requirement for informed consent because it used deidentified data.^15^ Results are reported in accordance with the Strengthening the Reporting of Observational Studies in Epidemiology (STROBE) reporting guideline cohort studies.^16^

### Study Population

We identified adults (aged at least 18 years) with diabetes enrolled in commercial non-HDHP employer-sponsored health plans between January 1, 2010, and December 31, 2018. Diabetes status was determined using Healthcare Effectiveness Data and Information Set (HEDIS) criteria.^17^ Race and ethnicity are classified in the OLDW as Asian, Hispanic, non-Hispanic Black (Black), or non-Hispanic White (White) based on self-report or derived rule sets based on name and zip code. Race and ethnicity were specifically assessed because Black and Hispanic patients with diabetes are significantly more likely to experience hypoglycemia- and hyperglycemia-related ED and hospital visits,^18,19^ and prior studies found lower rates of HSA enrollment (and potentially higher financial burden) among patients from minoritized racial and ethnic backgrounds, including Black and Hispanic individuals, compared with White individuals.^20,21^The first year during which a patient met HEDIS criteria and was enrolled in a non-HDHP was their baseline year. Our unit of analysis was the calendar year (eMethods in Supplement 1), and we required that patients have uninterrupted insurance coverage for each calendar year they were included. Each year after the first (baseline) year of non-HDHP coverage, enrollees could either remain in a non-HDHP or be forced to switch to an HDHP.

Patients were assigned to 1 of 2 groups. The HDHP group consisted of individuals who had at least 1 calendar year of enrollment in an HDHP following the baseline year (in a non-HDHP). We considered an individual to be enrolled in an HDHP during a calendar year if their in-network individual deductible on January 1 exceeded the minimum individual deductible qualifying them for a health savings account (HSA) in that year (eTable 1 in Supplement 1). All other individuals were considered to be enrolled in non-HDHPs. To reduce risks of selection and voluntary bias, we only included individuals whose employers did not offer a choice between HDHP and non-HDHP plans. Employers are identified in OLDW using a deidentified proxy variable, which allowed us to identify enrollees of employers that only offered an HDHP option. The non-HDHP group consisted of individuals who were never forced to switch into an HDHP. Individuals were censored at the end of the study period, disenrollment from their assigned health plan, or switching from an HDHP back to a non-HDHP, whichever came first.

### Independent Variables

Patient demographics were ascertained from OLDW enrollment files during the baseline year, including age, sex, race and ethnicity, US region of residency, and annual household income (see eMethods in Supplement 1). We captured all glucose-lowering medications filled during each calendar year (eTable 2 in Supplement 1), the baseline count of diabetes complications ascertained using the Diabetes Complications Severity Index,^22^ and the baseline year individual deductible amount. These covariates were selected based on their clinical relevance and association with severe dysglycemia.^18,19,23^

### Outcomes

ED visits and hospitalizations for severe hypoglycemia and hyperglycemia were identified using *International Classification of Diseases, Ninth Revision (ICD-9) *and* International Statistical Classification of Diseases and Related Health Problems, Tenth Revision *(*ICD-10*) diagnosis codes in the primary or first position in ED and hospital claims (eTable 3 in Supplement 1) as previously described.^18,19^ Results were quantified as the number of patients with at least 1 ED visit or hospitalization for hypoglycemia or hyperglycemia in a calendar year per 1000 persons (primary outcomes) and the number of hypoglycemic or hyperglycemic events requiring ED or hospital care per 1000 person-years (secondary outcomes).

Because only a fraction of severe hypoglycemic events^24,25^ and an unknown number of severe hyperglycemic events culminate in an ED or hospital encounter, and the decision to seek ED or hospital care may be influenced by health plan assignment, we secondarily examined having at least 1 office visit in a calendar year where severe hypoglycemia or hyperglycemia was coded and having any documented hypoglycemia or hyperglycemia (ie, office visit, ED visit, or hospitalization) in a calendar year. We did not count the total number of office visits because these do not correspond 1-to-1 to the total number of experienced events but rather signify that at least 1 event was experienced and subsequently coded during that encounter. All outcomes were captured separately for each calendar year in the study, including the baseline year.

### Statistical Analysis

For both groups, we calculated overall frequencies (percentages) and means (with SDs) for patient characteristics during the baseline year. Differences between groups were examined using standardized mean differences (SMDs) indicated as Cohen *d*. Cohen classified *d* values of 0.2, 0.5, and 0.8 as small, medium, and large effect sizes, respectively.^26^ For context, these *d* values approximately correspond to 15%, 33%, and 47% of nonoverlap between the 2 populations, respectively.^27,28,29^ We also calculated the mean annual individual and family deductibles and average outcome rates for all HDHP and non-HDHP periods separately.

To account for potential treatment selection bias, we constructed a propensity score for each individual by modeling the probability that, given their baseline characteristics, they would eventually switch to an HDHP.^30^ The propensity model included baseline age, sex, race and ethnicity, US region, annual household income, year (2009-2018), DCSI count, severe hypoglycemia and hyperglycemia, medications, and individual deductible, and was estimated using logistic regression. The estimated probabilities of switching to an HDHP derived from this model were used to construct inverse probability of treatment weights, which were then included in the outcomes models at the individual level to account for treatment selection.^30^

As appropriate for analyzing a repeated measures stepped wedge design, we estimated a series of mixed effects generalized linear models to examine the association between switching to an HDHP and the outcomes.^31^ Analyses were adjusted for baseline patient demographics (age, sex, race and ethnicity, US region, and annual household income), index year, severe hypoglycemia and hyperglycemia, and DCSI count. For each type of outcome (hypoglycemia, hyperglycemia) we estimated 4 primary models: (1) logistic model with the dependent variable of experiencing at least 1 event requiring ED or hospital care per year; (2) Poisson model with the dependent variable of the number of events requiring ED or hospital care; (3) logistic model with the dependent variable of any encounter (office, ED, or hospital); and (4) logistic model with the dependent variable of at least 1 office encounter. All models included random effects for the patient to account for multiple measurements per patient, elapsed years (0-10), and HDHP status. To assess whether HDHP status was associated with each of these outcomes, we tested whether the coefficient for HDHP status was nonzero. To assess whether the risk of each outcome increased with greater exposure to HDHP, we replicated these models using elapsed years of HDHP enrollment as the independent variable.

We conducted several sensitivity analyses. First, we included interaction terms between HDHP and race and ethnicity (patients from minoritized racial and ethnic groups vs White patients), and between HDHP and income (at least $40 000 vs less than $40 000) to examine whether patients from minoritized racial and ethnic groups and low-income patients experienced had different outcomes associated with switching to an HDHP. Second, the 4 models above were repeated including glucose-lowering medication classes as one of the adjustment covariates to examine the degree to which the association between HDHP and the outcome is mediated by the choice of glucose-lowering therapy.

For each model, we report the odds ratio (logit models) or risk ratio (count models); for years exposed, these represent average annual effects. We also report the adjusted annual HDHP and non-HDHP outcome rates and counts per 1000, assuming average values for all covariates. Two-sided *P* < .05 were considered statistically significant. All analyses were performed using Stata 16 (Stata Corp) and SAS version 7.1 (SAS Institute) from May 15, 2020, to November 3, 2022.

## Results

### Study Population

Our study population consisted of 245 055 enrollees, of whom 42 326 eventually switched to an HDHP and 202 729 remained in a non-HDHP (eFigure in Supplement 1). In the HDHP group, the mean (SD) age was 52 (10) years; 19 752 (46.7%) were women, 7375 (17.4%) were Black, 5740 (13.6%) were Hispanic, 26 572 (62.8%) were non-Hispanic White, and 6880 (16.3%) had annual household income less than $40 000 (Table 1). In the non-HDHP group, the mean (SD) age was 53 (10) years; 89 828 (44.3%) were women, 29 551 (14.6%) were Black, 26 689 (13.2%) were Hispanic, 130 843 (64.5%) were non-Hispanic White, and 28 288 (14.0%) had annual household income less than $40 000 (Table 1). Median individual and family deductible amounts while enrolled in HDHP and non-HDHP are shown in eTable 4 in Supplement 1. Crude numbers of hypoglycemia- and hyperglycemia-related ED visits or hospitalizations and office visits while enrolled in HDHP and non-HDHP are presented in eTable 5 in Supplement 1. The propensity model used to estimate the probability that a patient would switch had a *c*-statistic of 0.76.

**Table 1.** Baseline Patient Characteristics

Characteristic	Patients, No. (%)		SMD^a^
	HDHP group	Non-HDHP group	
No. of patients	42 326	202 729	NA
Age, y			
Mean (SD)	52 (10)	53 (10)	0.127
Median (range)	54 (18-85)	55 (18-86)	
18-44	8952 (21.2)	38 661 (19.1)	
45-64	31 188 (73.7)	144 959 (71.5)	
65-74	2022 (4.8)	17 741 (8.8)	
≥75	164 (0.4)	1368 (0.7)	
Sex			
Female	19 752 (46.7)	89 828 (44.3)	0.047
Male	22 574 (53.3)	112 901 (55.7)	
Race and ethnicity^b^			
Asian	1915 (4.5)	11 044 (5.4)	0.013
Black	7375 (17.4)	29 551 (14.6)	
Hispanic	5740 (13.6)	26 689 (13.2)	
White	26 572 (62.8)	130 843 (64.5)	
Other or unknown^c^	724 (1.7)	4602 (2.3)	
Region			
Midwest	8702 (20.6)	46 934 (23.2)	0.088
Northeast	2756 (6.5)	26 174 (12.9)	
South	25 014 (59.1)	100 586 (49.6)	
West	5828 (13.8)	28 708 (14.2)	
Unknown	26 (0.1)	327 (0.2)	
Annual household income, $			
<40 000	6880 (16.3)	28 288 (14.0)	0.097
40 000-74 999	12 198 (28.8)	54 714 (27.0)	
75 000-124 999	12 971 (30.6)	63 707 (31.4)	
125 000-199 999	5956 (14.1)	31 630 (15.6)	
≥200 000	3301 (7.8)	18 472 (9.1)	
Unknown	1020 (2.4)	5918 (2.9)	
Index year			
2010	18 113 (42.8)	57 067 (28.1)	0.462
2011	5754 (13.6)	23 933 (11.8)	
2012	4317 (10.2)	19 592 (9.7)	
2013	3280 (7.7)	14 881 (7.3)	
2014	4806 (11.4)	23 872 (11.8)	
2015	2309 (5.5)	15 486 (7.6)	
2016	2690 (6.4)	23 383 (11.5)	
2017	1042 (2.5)	18 468 (9.1)	
2018	15 (0.0)	6047 (3.0)	
Baseline deductible amount, $			
0	4650 (11.0)	60 839 (30.0)	0.707
<250	4867 (11.5)	37 334 (18.4)	
251-500	13 116 (31.0)	69 047 (34.1)	
501-1350	19 693 (46.5)	35 509 (17.5)	
Diabetes complications count			
0	26 910 (63.6)	124 176 (61.3)	0.059
1	10 251 (24.2)	50 231 (24.8)	
2	3430 (8.1)	18 311 (9.0)	
3	1192 (2.8)	6821 (3.4)	
4	403 (1.0)	2342 (1.2)	
5	118 (0.3)	723 (0.4)	
6	22 (0.1)	125 (0.1)	
Hypoglycemia ED visits or hospitalizations	1092 (2.6)	5165 (2.5)	0.002
Hyperglycemia ED visits or hospitalizations	207 (0.5)	891 (0.4)	0.007
Glucose-lowering medications			
α-Glucosidase inhibitors	111 (0.3)	545 (0.3)	0.001
Amylin analogs	141 (0.3)	481 (0.2)	0.018
DPP-4 inhibitor	6176 (14.6)	28 634 (14.1)	0.013
Glinide	301 (0.7)	1590 (0.8)	0.008
GLP1-receptor agonist	2935 (6.9)	15 042 (7.4)	0.019
Insulin	9392 (22.2)	42 591 (21.0)	0.029
Metformin	23 310 (55.1)	109 022 (53.8)	0.026
SGLT-2 inhibitors	962 (2.3)	8093 (4.0)	0.099
Sulfonylureas	10 964 (25.9)	47 193 (23.3)	0.061
Thiazolidinedione	5599 (13.2)	21 418 (10.6)	0.082
No fills for any glucose-lowering medications	10 473 (24.7)	54 525 (26.9)	0.049

Abbreviations: DPP-4, dipeptidyl-peptidase 4; GLP-1, glucagon-like peptide-1; HDHP, high-deductible health plans; NA, not applicable; SGLT-2, sodium-glucose cotransporter 2; SMD, standardized mean difference.

^a^ Differences between groups were examined using SMDs indicated as Cohen *d.* Cohen classified *d* values of 0.2, 0.5, and 0.8 as small, medium, and large effect sizes, respectively.^26^ For context, these *d* values approximately correspond to 15%, 33%, and 47% of nonoverlap between the 2 populations, respectively.^27,28,29^

^b^ Race is classified in the OptumLabs Data Warehouse database as Asian, Hispanic, non-Hispanic Black (Black), or non-Hispanic White (White) based on self-report or derived rule sets based on name and zip code.

^c^ Other is a racial and ethnicity choice in the OptumLabs Data Warehouse database.

### Severe Hypoglycemia After Switching to an HDHP

Switching to an HDHP did not change the odds of experiencing any hypoglycemia-related ED visit or hospitalization when comparing all study years (OR, 1.01 [95% CI, 0.95-1.06]; *P* = .85), but each year of HDHP enrollment increased the odds of hypoglycemia-related ED or hospital care by 2% (OR, 1.02 [95% CI, 1.00-1.04]; *P* = .04) (Table 2). The odds of experiencing any severe hypoglycemic event were significantly higher among the HDHP group (OR, 1.11 [95% CI, 1.08-1.14]; *P* < .001), corresponding to 311 per 1000 HDHP enrollees per year having any coded hypoglycemia, compared with 282 per 1000 non-HDHP enrollees per year. Each year of HDHP enrollment increased these odds by 5% (OR, 1.05 [95% CI, 1.04-1.06]; *P* < .001). Finally, the proportion of HDHP enrollees with at least 1 office visit where hypoglycemia was coded was 14% higher than among non-HDHP enrollees (OR, 1.14 [95% CI, 1.11-1.17]; *P* < .001), with each year of HDHP enrollment increasing these odds by 6% (OR, 1.06 [95% CI, 1.05-1.07]; *P* < .001).

**Table 2.** Association Between Switching to an HDHP and Severe Hypoglycemia^a^

Event	Odds ratio (95% CI)	Estimated counts per 1000 person-years^b^		*P* value
		HDHP group	Non-HDHP group	
Any ED visit or hospitalization	1.01 (0.95-1.06)	51	51	.85
Years in HDHP	1.02 (1.00-1.04)	51	50	.04
Cumulative count ED visits and hospitalizations	1.01 (0.96-1.07)^c^	45	44	.67
Years in HDHP	1.03 (1.01-1.05)	45	43	.004
Any office visit	1.14 (1.11-1.17)	222	195	<.001
Years in HDHP	1.06 (1.05-1.07)	205	194	<.001
Any event	1.11 (1.08-1.14)	311	282	<.001
Years in HDHP	1.05 (1.04-1.06)	294	280	<.001

Abbreviations: ED, emergency department; HDHP, high-deductible health plan.

^a^ Multivariable regression examined the association between switching to an HDHP and 4 hypoglycemia outcomes of interest: at least 1 ED or hospitalization (primary outcome), the cumulative count of ED visits and hospitalizations, at least 1 office visit, and at least 1 event in any setting. Secondary analyses examined the association of the number of years enrolled in an HDHP with each of the outcomes. Models are adjusted for patient demographics (age, sex, race and ethnicity, US region, and annual household income), index year, history of severe hypoglycemia or hyperglycemia requiring ED or hospital care, and baseline count of diabetes complications.

^b^ Outcomes of any events are presented as the number of people per 1000 who had at least 1 event during a calendar year. The cumulative number of ED visits and hospitalizations is presented as the number of events experienced per 1000 people per calendar year.

^c^ Risk ratio was calculated for count outcomes.

Results were unchanged with adjustment for glucose-lowering medications (eTable 6 in Supplement 1). When we included an interaction term between switching to HDHP and annual household income, we found that the change in hypoglycemia-related ED and hospital visits associated with switching to an HDHP was smaller among patients making more than $40 000 compared with those making less than $40 000 (interaction OR, 0.76 [95% CI, 0.67-0.87]; *P* < .001). The change in office visits for hypoglycemia, however, was the same among patients with high income and low income. There was no difference in the change in hypoglycemia-related ED or hospital visits associated with switching to an HDHP as a function of race and ethnicity (interaction *P* = .22), but the change in office visits was smaller among patients from minoritized racial and ethnic groups compared with White patients (interaction OR, 0.91 [95% CI, 0.86-0.96]; *P* < .001).

### Severe Hyperglycemia After Switching to an HDHP

Switching to an HDHP was associated with a significant increase in the odds of experiencing at least 1 hyperglycemia-related ED or hospital visit annually (OR, 1.25 [95% CI, 1.11-1.42]; *P* < .001) (Table 3). Each year of HDHP enrollment increased these odds by 5% (OR, 1.05 [95% CI, 1.01-1.09]; *P* = .02). There was no association between forced switch to HDHP and having hyperglycemia-related office visits or any severe hyperglycemia.

**Table 3.** Association Between Switching to an HDHP and Severe Hyperglycemia^a^

Event	Odds ratio (95% CI)	Estimated counts per 1000 person-years^b^		*P* value
		HDHP group	Non-HDHP group	
Any ED visit/hospitalization	1.25 (1.11-1.42)	5	4	<.001
Years in HDHP	1.05 (1.01-1.09)	4	4	.02
Cumulative count of ED visits/hospitalizations	1.30 (1.15-1.47)^c^	3	3	<.001
Years in HDHP	1.05 (1.01-1.09)	3	3	.01
Any office visit	0.99 (0.93-1.05)	11	12	.72
Years in HDHP	1.00 (0.99-1.02)	12	11	.69
Any event	1.03 (0.98-1.09)	19	18	.24
Years in HDHP	1.01 (1.00-1.03)	18	18	.16

Abbreviations: ED, emergency department; HDHP, high-deductible health plan.

^a^ Multivariable regression examined the association between switching to an HDHP and 4 hypoglycemia outcomes of interest: at least 1 ED or hospitalization (primary outcome), the cumulative count of ED visits and hospitalizations, at least 1 office visit, and at least 1 event in any setting. Secondary analyses examined the association of the number of years enrolled in an HDHP with each of the outcomes. Models are adjusted for patient demographics (age, sex, race and ethnicity, US region, and annual household income), index year, history of severe hypoglycemia or hyperglycemia requiring ED or hospital care, and baseline count of diabetes complications.

^b^ Outcomes of any events are presented as the number of people per 1000 who had at least one event during a calendar year. The cumulative number of ED visits/hospitalizations is presented as the number of events experienced per 1000 people per calendar year.

^c^ Risk ratio was calculated for count outcomes.

Results were unchanged with adjustment for glucose-lowering medications (eTable 7 in Supplement 1). There was no significant interaction between switching to an HDHP and race and ethnicity with respect to either hyperglycemia-related ED or hospital visits or office visits, or between switching to an HDHP and income with respect to hyperglycemia-related ED or hospital visits. However, switching to an HDHP was associated with a slightly bigger change in hyperglycemia-related office visits among those making at least $40 000 compared with those making less than $40 000 (interaction OR, 1.17 [95% CI, 1.00-1.37]; *P* = .047).

## Discussion

Severe hypoglycemic and hyperglycemic events are associated with substantial morbidity,^32^ mortality,^33,34^ and impaired quality of life.^35,36,37,38,39^ For adults living with diabetes, each year of enrollment in HDHP increased their odds of experiencing an ED or hospital visit for hypoglycemia by 2% and for hyperglycemia by 5%, with additional evidence of deferred ambulatory care focused on these events, reflecting missed opportunities to improve glycemic control. Because severe dysglycemic events may be prevented with optimal glycemic management, the increase in the frequency of their occurrence suggests important gaps in access to and implementation of diabetes therapy.

People living with diabetes face high and growing out-of-pocket expenses for managing their disease.^40^ HDHPs require that all deductible limits are paid before insurance begins to cover eligible services, which at a minimum was $1400 for an individual in 2020 through 2022; yet, 4 in 10 US residents may not be able to cover even a $400 emergency expense.^41^ As a result, individuals may be forced to ration medications, glucose monitoring supplies, diabetes self-management education, food, and other essential cares^42^ to the detriment of their health. In prior studies, both hypoglycemia and hyperglycemia have been associated with low-income^19,43,44,45^ and food insecurity,^43,45,46,47,48^ and our analyses found that forced transition to HDHP similarly increases these risks.

Rationing of medications and/or testing supplies can directly precipitate severe hyperglycemia, which explains the bigger changes observed with hyperglycemia-related ED and hospital visits associated with forced HDHP switches compared with hypoglycemia-related ED and hospital visits. Severe hyperglycemia is also less likely to be treated successfully in the ambulatory setting,^49,50^ such that patients experiencing these events would likely be unable to defer seeking emergency medical care even when facing financial hardship. However, once discharged from the ED or hospital, HDHP enrollees may hesitate to follow-up and discuss these events in the office, which would manifest as comparable rates of hyperglycemia-related office visits but higher rates of ED and hospital visits among HDHP enrollees compared with non-HDHP enrollees. This missed opportunity to optimize glycemic control in the ambulatory setting may further predispose patients to recurrent events.

Our data also found increased rates of severe hypoglycemia—evidenced by higher rates of hypoglycemia-related office visits—upon switching to HDHP, which may also be caused by rationing testing supplies, food, and routine diabetes care. Importantly, this is an underestimate of the increase in hypoglycemic events that likely occurred with the transition to HDHPs, because—as for severe hyperglycemia—HDHP enrollees may forego ambulatory care for cost-related reasons just as they forego other care. Because patients and caregivers may be able to treat hypoglycemia at home, the increased frequency of hypoglycemia subsequent to HDHP transition is unlikely to directly translate to higher rates of hypoglycemia-related ED and hospital encounters. While in principle consistent with the mission of HDHPs of shifting care to lower cost settings, in this case, the increased frequency of severe hypoglycemia—no matter where managed and discussed—is a sign of detrimental effects of HDHP enrollment for people living with diabetes. In addition, foregoing medical care for severe hypoglycemic events is associated with greater likelihood of their recurrence.^24^

We were not surprised to find that forced switch to HDHP was associated with more hypoglycemia-related ED and hospital visits among patients with lower income, which is consistent with prior studies of diabetes and other health conditions.^7,8,10,13,51^ There was no interaction between income and hypoglycemia-related office visits, raising concerns about rationing of what patients may perceive to be elective care if patients with low income experience severe hypoglycemia requiring ED and hospital care but then forego ambulatory care to improve their glycemic control. Similarly, while patients with low income were as likely as patients with high income to see an increase in hyperglycemia-related ED or hospital visits after switching to HDHP, low-income patients were less likely to see an increase in hyperglycemia-related office visits. Thus, although all patients may need to seek urgent medical care for severe hyperglycemia, patients with low income are more likely to forego ambulatory care to discuss these events, resulting in missed opportunities to improve glycemic control.

We did not find significant differences in the changes for hypoglycemia- or hyperglycemia-related ED and hospital visits associated with switching to HDHP between patients with diabetes from minoritized racial and ethnic groups and White patients with diabetes. However, patients from minoritized racial and ethnic groups were less likely than White patients to see an increase in hypoglycemia-related office visits, suggesting a deferral of ambulatory care that could improve glycemic control. When considered in the context of earlier work showing that Black and Hispanic individuals experience hypoglycemia-related ED and hospital visits more often than White individuals,^18^ these findings underscore the concern that patients from minoritized racial and ethnic groups are foregoing needed care. Indeed, prior studies have found that individuals from minoritized racial and ethnic groups are less likely to participate in HSAs than White individuals,^20,21^ which can exacerbate cost-related barriers to care. However, additional research is needed to fully understand the impact of health benefit design on racial and ethnic disparities in care delivery, accessibility, and health outcomes as well as why we did not see similar differences between individuals from minoritized racial and ethnic groups and White individuals for severe hyperglycemia.

Given the detrimental health outcomes associated with HDHPs, employers need to be more judicious in their recommendation of health plan options for individuals and families with diabetes and other chronic health conditions that require ongoing care. Employers should offer a variety of health plan options to their employees and be transparent about the full costs associated with each plan in different clinical scenarios. Previous studies have shown that enrollees are not fully aware of the details within their health plans and may be focusing on reducing the cost of monthly premiums—not overall care—when choosing health plans.^52^ Requiring employers to fund HSAs, particularly for individuals with low income, may help mitigate some of the cost-related barriers to seeking medical care, as patients with low income are generally less likely than patients with high income to participate in HSAs^20,21^ and HSA enrollment has been shown to mitigate some of the negative outcomes of HDHP enrollment.^21^

HDHP redesign may also reduce their detrimental health outcomes. Some employers and health plans have started to develop value-based insurance designs, such as preventive drug lists, that would encourage patients to use high-value services at a lower cost.^53^ In 2019, the Internal Revenue Service has expanded preventative care and chronic disease exemptions to HSA-qualifying deductibles to include diabetes medications (insulin and other glucose-lowering agents, statins, angiotensin-converting enzyme inhibitors), retinopathy screening, glucose meters, and hemoglobin A_1C_ testing,^54^ which allows these services to be covered by insurance before the full deductible is met. Recent efforts to cap the cost of insulin may also mitigate the detrimental health outcomes associated with HDHPs.

### Limitations

This study had some limitations. As an observational study, it is not possible to make causal inferences or elucidate the reasons why patients enrolled in HDHPs experience more severe hypoglycemic and hyperglycemic events. It will be important, in future research, to directly engage people living with diabetes who are forced to switch to HDHPs to understand their experiences and the impact on diabetes self-management, cost-related nonadherence, food insecurity, and more. Enrollees may have switched plans for reasons that are not observed but that increase the risk of severe dysglycemia. Although we sought to reduce this bias by only including patients with employer-forced switches, misclassification of this variable in OLDW is possible. We could not examine whether HDHP enrollees had HSA accounts or whether these accounts were funded by their employer. However, an employer-funded HSA would defray some of the financial burden of high deductibles and bias our findings toward the null. We also excluded patients with less than 2 years of uninterrupted insurance coverage, who because of interruptions in employment or insurance coverage may be most susceptible to the adverse outcomes of HDHP enrollment.

Because our objective was to follow patients longitudinally and patients entered the study when eligibility criteria were first met (in order to minimize risk of bias), the majority of the study population entered the cohort before 2013. Additionally, employers could have offered employees a choice between an HDHP observed in OLDW and a non-HDHP from a different insurance carrier; the latter would be missing from our data. Our findings apply to situations where employers only offer an HDHP option and not where employees have a range of options to choose from. Because we did not have access to employer characteristics, which may influence their choice of health plan offerings, there is potential for unmeasured confounding if employer characteristics are also associated with employee’s likelihood of experiencing severe dysglycemic events. Additionally, our data cannot capture events managed outside the health care system that do not generate a claim, thereby underestimating the detrimental impact of HDHPs.

## Conclusions

Although HDHPs were created to lower insurance premiums and promote cost savings by limiting low value care, their implementation has adversely affected health outcomes in patients with diabetes. Employer-mandated switching to an HDHP was associated with increased odds of severe hypoglycemic and hyperglycemic events, which increased with each year of HDHP enrollment. Individuals with low income and from minoritized racial and ethnic groups were especially susceptible to the detrimental outcomes of HDHP transition. Thus, HDHP enrollees may be rationing or foregoing necessary care, which is detrimental to their health and ultimately increases the morbidity, mortality, and costs associated with diabetes.

## Notes

Supplement 1. **eMethods.** Supplementary Methods **eTable 1.** Minimum Deductible to Qualify for a Health Savings Account Limit, as Defined by the Internal Revenue Service **eTable 2.** List of Glucose-Lowering Medications Included **eTable 3.** International Classification of Disease (ICD)-9 and ICD-10 Codes for Severe Hyperglycemia and Severe Hypoglycemia **eTable 4.** Annual Deductible Amounts Among Enrollees in HDHP and non-HDHP Health Plans **eTable 5.** Crude Rates of Having Any Emergency Department, Hospital, and Office Visits for Severe Hypoglycemia and Hyperglycemia While Enrolled in a HDHP or non-HDHP **eTable 6.** Association Between HDHP and Severe Hypoglycemia With Medication Adjustment **eTable 7.** Association Between HDHP and Severe Hyperglycemia With Medication Adjustment **eFigure.** Flow Chart
eReferences

Supplement 2.
Data Sharing Statement
