# Supplementary material for: Evaluation of High-Deductible Health Plans and Acute Glycemic Complications Among Adults With Diabetes
Source: JAMA Netw Open. 2023 Jan 20;6(1):e2250602. doi: 10.1001/jamanetworkopen.2022.50602 (PMC9860518; doi:10.1001/jamanetworkopen.2022.50602)
Supplement: Supplement 1. — eMethods. Supplementary Methods eTable 1. Minimum Deductible to Qualify for a Health Savings Account Limit, as Defined by the Internal Revenue Service eTable 2. List of Glucose-Lowering Medications Included eTable 3. International Classification of Disease (ICD)-9 and ICD-10 Codes for Severe Hyperglycemia and Severe Hypoglycemia eTable 4. Annual Deductible Amounts Among Enrollees in HDHP and non-HDHP Health Plans eTable 5. Crude Rates of Having Any Emergency Department, Hospital, and Office Visits for Severe Hypoglycemia and Hyperglycemia While Enrolled in a HDHP or non-HDHP eTable 6. Association Between HDHP and Severe Hypoglycemia With Medication Adjustment eTable 7. Association Between HDHP and Severe Hyperglycemia With Medication Adjustment eFigure. Flow Chart eReferences [file jamanetwopen-e2250602-s001.pdf]

Supplemental Online Content

Jiang DH, Herrin J, Van Houten HK, McCoy RG. Evaluation of high-deductible health plans and acute glycemic complications among adults with diabetes. *JAMA Netw Open.* 2023;6(1):e2250602. doi:10.1001/jamanetworkopen.2022.50602

eMethods. Supplementary Methods

eTable 1. Minimum Deductible to Qualify for a Health Savings Account Limit, as Defined by the Internal Revenue Service

eTable 2. List of Glucose-Lowering Medications Included

eTable 3. International Classification of Disease (ICD)-9 and ICD-10 Codes for Severe Hyperglycemia and Severe Hypoglycemia

eTable 4. Annual Deductible Amounts Among Enrollees in HDHP and non-HDHP Health Plans

eTable 5. Crude Rates of Having Any Emergency Department, Hospital, and Office Visits for Severe Hypoglycemia and Hyperglycemia While Enrolled in a HDHP or non-HDHP

eTable 6. Association Between HDHP and Severe Hypoglycemia With Medication Adjustment

eTable 7. Association Between HDHP and Severe Hyperglycemia With Medication Adjustment

eFigure. Flow Chart

eReferences

This supplemental material has been provided by the authors to give readers additional information about their work.

© 2023 Jiang DH et al. *JAMA Network Open.*

eMethods. Supplementary Methods

Study Design

This was a stepped wedge study design, where each patient who switched to a high-deductible health plan (HDHP) served as their own control, while those who never switched served as contemporaneous controls. Accordingly, we used analytic methods appropriate for a stepped wedge design with repeated measures on individuals. While stepped wedge designs are typically randomized, they are also an appropriate framework for observational studies when the intervention is staggered over time and the followup time varies.[1-3]

We chose to use calendar year as the unit of observation for several reasons. First, our primary outcome, ED or hospital visits related to diabetes complications, had a crude event rate of 1%-2% each year, and shorter time periods would made modelling such events challenging. Second, we anticipated that the effect of switching health plans would not be immediately measurable; rather, that if higher deductibles led to reduced or deferred care this would lead to diabetes complications-related visits over longer periods of time, such as a year. Third, longer observation periods (e.g., pre and post design pooling all unexposed and exposed time) would not easily allow us to incorporate multiple time periods per individual, nor would we be able to align observations across exposed and unexposed groups. Using calendar year as our unit of observation allows us to treat each year as the 'step' of a stepped wedge design and compare exposed and unexposed individuals directly.

Socioeconomic Data in OLDW

Our study included race/ethnicity and annual household income as independent variables. Race is classified in OptumLabs® Data Warehouse (OLDW) as non-Hispanic White (White), non-Hispanic Black (Black), Asian, Hispanic, or other based on self-report or imputation by the data provider using data such as full name and ZIP code.[4] Annual household income included in OLDW is sourced from a national supplier of consumer marketing data; it is imputed by the data supplier based on a model that uses both public and private consumer data such as credit card statements, loan amounts, and loan payments. A household is defined as individuals with the same last name living at the same street address.[4]

© 2023 Jiang DH et al. *JAMA Network Open.*

eTable 1. Minimum Deductible to Qualify for a Health Savings Account Limit, as Defined by the Internal Revenue Service.

Year	Minimum Individual Deductible to Qualify	Minimum Family Deductible to Qualify
2010	$1,200.00	$2,400.00
2011	$1,200.00	$2,400.00
2012	$1,200.00	$2,400.00
2013	$1,250.00	$2,500.00
2014	$1,250.00	$2,500.00
2015	$1,300.00	$2,600.00
2016	$1,300.00	$2,600.00
2017	$1,300.00	$2,600.00
2018	$1,350.00	$2,700.00

© 2023 Jiang DH et al. *JAMA Network Open.*

eTable 2. List of Glucose-lowering Medications Included

Medication Class	Included Agents
**Alpha-glucosidase inhibitors**	Acarbose
	Miglitol
**Amylin**	Pramlintide
**Dipeptidyl peptidase-4 (DPP-4) inhibitors**	Alogliptin
	Linagliptin
	Sitagliptin
	Saxagliptin
**Glinides**	Nateglinide
	Repaglinide
**Thiazolidinediones**	Pioglitazone
	Rosiglitazone
	Troglitazone
**Glucagon-like peptide-1 (GLP-1) receptor agonists**	Exenatide
	Liraglutide
	Albiglutide
	Dulaglutide
	Semaglutide
	Lixisenatide
**Insulin: Bolus-Human**	Regular
**Insulin: Bolus-Analog**	Aspart
	Lispro
	Glulisine
	Inhaled powder insulin
**Insulin: Basal-Human**	Neutral Protamine Hagedorn
	Isophane
**Insulin: Basal-Analog**	Detemir
	Glargine
	Degludec
**Biguanide**	Metformin
**Sodium-glucose transport protein 2 (SGLT-2) inhibitors**	Canagliflozin
	Empagliflozin
	Dapagliflozin
	Ertugliflozin
**Sulfonylureas**	Acetohexamide
	Chlorpropamide
	Glimepiride
	Glipizide
	Glyburide
	Tolazamide
	Tolbutamide

© 2023 Jiang DH et al. *JAMA Network Open.*

eTable 3. International Classification of Disease (ICD)-9 and ICD-10 Codes for Severe Hyperglycemia and Severe Hypoglycemia

**Severe hypoglycemia**	ICD-9-CM codes[5] 251.0, 251.1, 251.2, 962.3, and 250.8x (unless one of the following codes also appears on the same claim as 250.8x: 259.8, 272.7, 681.x, 682.x, 686.9, 707.1x, 707.2x, 707.8, 707.9, 709.3, 730.0x, 730.1x, 730.2x, 731.8).<br><br>ICD-10-CM codes E10.641, E10.649, E11.641, E11.649, E13.641, E13.649, E16.0, E16.1, E16.2, T38.3X1A, T38.3X1D, T38.3X1S, T38.3X2A, T38.3X2D, T38.3X2S, T38.3X3A, T38.3X3D, T38.3X3S, T38.3X4A, T38.3X4D, T38.3X4S, T38.3X5A, T38.3X5D, and T38.3X5S
**Severe hyperglycemia**	ICD-9-CM codes 250.1x and 250.2x<br><br>ICD-10-CM codes E10.10, E10.11, E11.00, E11.01, E11.10, E11.11, E13.00, E13.01, E13.10, and E13.11

© 2023 Jiang DH et al. *JAMA Network Open.*

eTable 4. Annual deductible amounts among enrollees in HDHP and non-HDHP health plans.

	**HDHP Median (IQR)**	**Non-HDHP Median (IQR)**
**Individual**	1500 [1250, 2000]	350 [50, 500]
**Family**	3000 [2500, 3500]	800 [0, 1400]

© 2023 Jiang DH et al. *JAMA Network Open.*

eTable 5. Crude rates of having any emergency department, hospital, and office visits for severe hypoglycemia and hyperglycemia while enrolled in a HDHP or non-HDHP.

	**HDHP**<br>% (person-years with event / person-years of observation)	**Non-HDHP**<br>% (person-years with event / person-years of observation)
**Severe Hypoglycemia**		
ED visits/hospitalization	4.1 (527/128,551)	4.0 (3930/975,666)
Office	24.6 (3158/128,551)	22.8 (22,278/975,666)
**Severe Hyperglycemia**		
ED visits/hospitalization	0.8 (102/128,551)	0.6 (609/975,666)
Office	5.0 (637/128,551)	4.1 (4049/975,666)

© 2023 Jiang DH et al. *JAMA Network Open.*

eTable 6. Association between HDHP and severe hypoglycemia with medication adjustment.
Multivariable regression examined the association between switching to a HDHP and four hypoglycemia outcomes of interest: at least one ED/hospitalization (primary outcome), the cumulative count of emergency department (ED) visits/hospitalizations, at least one office visit, and at least one event in any setting. Secondary analyses examined the impact of the number of years enrolled in a HDHP on each of the outcomes. Models are adjusted for patient demographics (age, sex, race/ethnicity, U.S. region, and annual household income), index year, baseline history of severe hypoglycemia or hyperglycemia, baseline count of diabetes complications, and glucose-lowering medications.

	**Odds Ratio**[a]	**Estimated Counts**[b] **per 1,000 person years**		**p-value**
		**HDHP Group**	**Non-HDHP Group**	
Any ED Visit /Hospitalization	0.97 (0.92, 1.03)	58	60	0.29
Year HDHP	1.00 (0.98, 1.02)	59	59	0.83
Cumulative Count ED Visits/Hospitalizations	1.00 (0.95, 1.05) [a]	54	54	0.84
Year HDHP	1.01 (0.99, 1.03)	54	54	0.23
Any Office Visit	1.11 (1.08, 1.15)	238	213	<0.001
Year HDHP	1.04 (1.03, 1.05)	223	213	<0.001
Any Event	1.08 (1.06, 1.11)	341	315	<0.001
Year HDHP	1.04 (1.03, 1.05)	325	314	<0.001

Abbreviations: ED, emergency department; HDHP, high deductible health plan.
[a] Risk ratio was calculated for count outcomes
[b] Outcomes of "any" events are presented as the number of people per 1000 who had at least one event during a calendar year. The cumulative number of ED visits/hospitalizations is presented as the number of events experienced per 1000 people per calendar year.

© 2023 Jiang DH et al. *JAMA Network Open.*

eTable 7. Association between HDHP and severe hyperglycemia with medication adjustment

Multivariable regression examined the association between switching to a HDHP and four hyperglycemia outcomes of interest: the cumulative count of emergency department (ED) visits/hospitalizations, at least one ED/hospitalization (primary outcome), the cumulative count of emergency department (ED) visits/hospitalizations, at least one office visit, and at least one event in any setting. Secondary analyses examined the impact of the number of years enrolled in a HDHP on each of the outcomes. Models are adjusted for patient demographics (age, sex, race/ethnicity, U.S. region, and annual household income), index year, baseline history of severe hypoglycemia or hyperglycemia, baseline count of diabetes complications, and glucose-lowering medications.

	Odds Ratio[a]	Estimated Counts[b] per 1,000 person years		p-value
		HDHP Group	Non-HDHP Group	
Any ED Visit/Hospitalization	1.21 (1.07, 1.37)	5	4	0.002
Year HDHP	1.04 (1.00, 1.08)	5	5	0.07
Cumulative Count ED Visits/Hospitalizations	1.26 (1.11, 1.41) [a]	4	3	<0.001
Year HDHP	1.04 (1.00, 1.08)	4	3	<0.001
Any Office Visit	0.97 (0.92, 1.03)	11	12	0.36
Year HDHP	1.00 (0.98, 1.02)	12	12	0.81
Any Event	1.02 (0.96, 1.07)	19	19	0.56
Year HDHP	1.01 (0.99, 1.02)	19	19	0.54

Abbreviations: ED, emergency department; HDHP, high deductible health plan.
[a] Risk ratio was calculated for count outcomes
[b] Outcomes of "any" events are presented as the number of people per 1000 who had at least one event during a calendar year. The cumulative number of ED visits/hospitalizations is presented as the number of events experienced per 1000 people per calendar year.

© 2023 Jiang DH et al. *JAMA Network Open.*

eFigure. Flow Chart

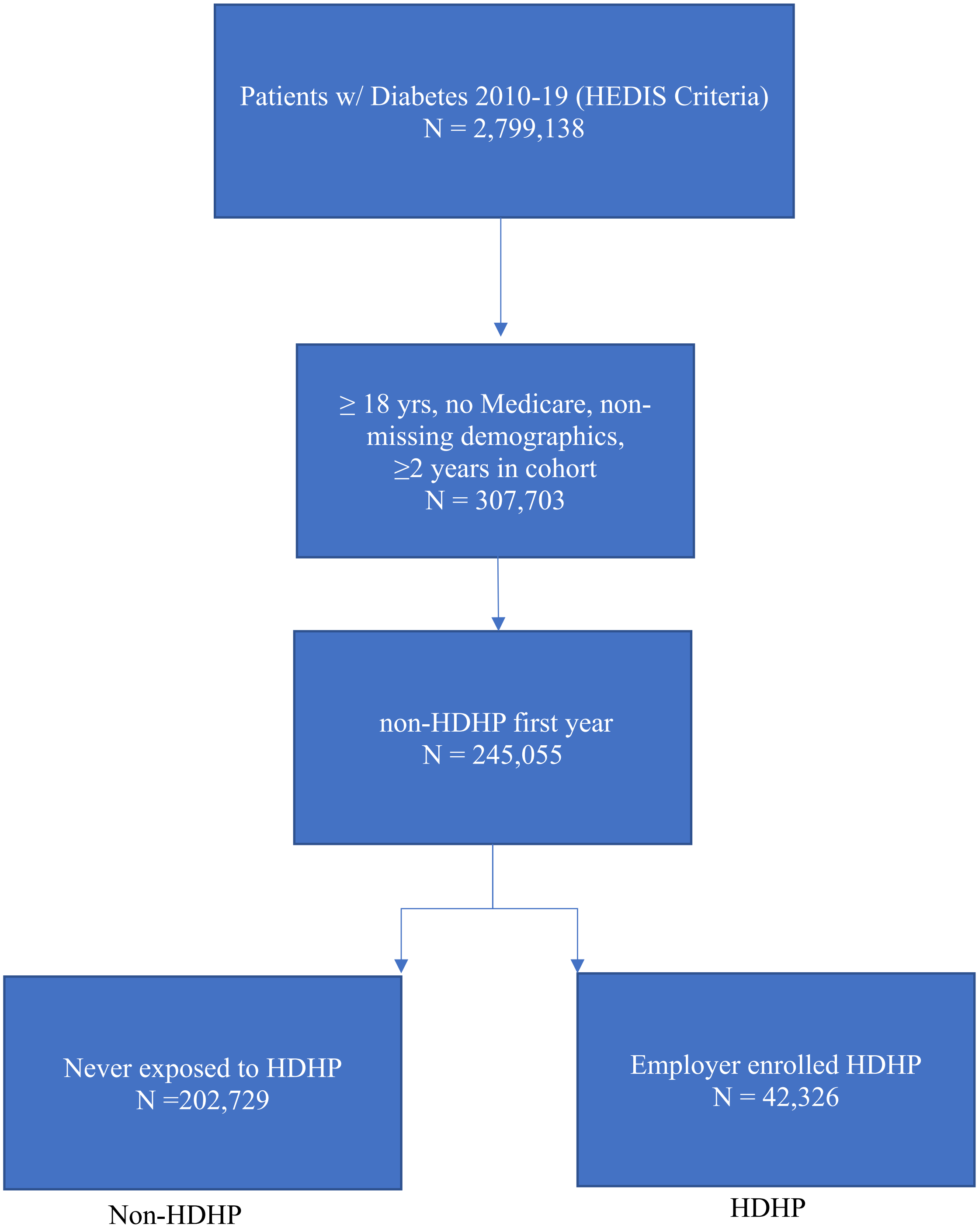

© 2023 Jiang DH et al. *JAMA Network Open.*

eReferences

1. Cook TD, Campbell DT. *Quasi-experimentation: Design and analysis issues for field settings.* Boston, MA, USA: Houghton Mifflin; 1979.
2. Worster DT, Franke MF, Bazua R, et al. Observational stepped-wedge analysis of a community health worker-led intervention for diabetes and hypertension in rural Mexico. *BMJ Open.* 2020;10(3):e034749.
3. Herrin J, da Graca B, Nicewander D, et al. The effectiveness of implementing an electronic health record on diabetes care and outcomes. *Health Serv Res.* 2012;47(4):1522-1540.
4. Hershman DL, Tsui J, Wright JD, Coromilas EJ, Tsai WY, Neugut AI. Household net worth, racial disparities, and hormonal therapy adherence among women with early-stage breast cancer. *J Clin Oncol.* 2015;33(9):1053-1059.
5. Ginde AA, Blanc PG, Lieberman RM, Camargo CA, Jr. Validation of ICD-9-CM coding algorithm for improved identification of hypoglycemia visits. *BMC Endocr Disord.* 2008;8:4.

© 2023 Jiang DH et al. *JAMA Network Open.*
