# Supplementary material for: Evaluation of High-Deductible Health Plans and Acute Glycemic Complications Among Adults With Diabetes
Source: JAMA Netw Open. 2023 Jan 20;6(1):e2250602. doi: 10.1001/jamanetworkopen.2022.50602 (PMC9860518; doi:10.1001/jamanetworkopen.2022.50602)
Supplement: Supplement 2. — Data Sharing Statement [file jamanetwopen-e2250602-s002.pdf]

Data Sharing Statement

Jiang. Evaluation of High-Deductible Health Plans and Acute Glycemic Complications Among Adults With Diabetes. *JAMA Netw Open*. Published January 20, 2023.
doi:10.1001/jamanetworkopen.2022.50602

Data
Data available: No

Additional Information
Explanation for why data not available: This study was conducted using deidentified claims data from OptumLabs Data Warehouse, which is only available to researchers within partner organizations as part of approved studies.
